# Dilatation of Posterior Cerebral Vessels with Basal Hemorrhage in a Girl Aged Sixteen
1Read at a meeting of the Bristol Medico-Chirurgical Society, November 11th, 1908.


**Published:** 1908-12

**Authors:** T. M. Carter, I. Walker Hall


### DILATATION OF POSTERIOR CEREBRAL VESSELS WITH BASAL HEMORRHAGE IN A GIRL AGED SIXTEEN.[1]

BY

T. M. Carter, M.D.

(*With remarks by I. Walker Hall, M.D.*)

---

Basal hemorrhage occurring in persons under 30 years of age is not a rare though by no means a common condition. Its liability to be overlooked or mistaken for functional disturbance has recently been commented upon by Professor Rose Bradford.[2]

These cases occur at any age from early childhood to extreme old age, but chiefly between 20 and 30 years, where an aneurysm, usually sacculated and varying in size from that of a pin's head to a large walnut, is formed in one of the vessels at the base of the brain, usually the middle cerebral or the basilar: slight leaking occurs, and gives rise to a train of indefinite symptoms, in which there is at first usually a kind of fit, or faint, or seizure, with, it may be, loss of consciousness for but a short time, followed by a varying period of indefinite illness, often very slight, and characterised by vomiting, giddiness, disturbances of vision, tinnitus, lassitude, generally loss of knee-jerks, and

[1] Read at a meeting of the Bristol Medico-Chirurgical Society, November 11th, 1908.

[2] Vide *Lancet*, 1908, ii. 703.

often accompanied by more or less stiffness and rigidity of the muscles of the neck: then suddenly a fatal seizure from rupture of the aneurysm supervenes.

The course followed by the extravasated blood is determined by the pia-mater; rarely does this rupture and a subdural hemorrhage result: a large quantity of blood may be found in the lateral ventricles, which has all come from the vessel ruptured at the base and passed up the side of the pons and medulla into the fourth ventricle, and so to the third and the lateral ventricles.

The pathological findings in a case of this type, which came under my notice in September, contained features sufficiently unusual to merit a permanent record.

The patient was a servant-girl, Charlotte A——, aged 16, but with the appearance and development of a young woman of 20, somewhat fat and very anæmic. She had been engaged by her mistress seven weeks previously without much inquiry as to her health and antecedents, and had complained of headaches, noises in the head, some deafness, indefinite pains in the back and legs, and indigestion during this time, her mistress making the significant observation that she did not think she was really deaf, but slow and dull, for she had noticed after a while that it was never necessary for her to repeat an order, the girl would "wake up to it" in time. Sometimes she was giddy, and on two occasions she had fallen on the stairs, but it was at a dark corner where others also occasionally fell.

I learned from her mother after the girl's death that she had seen her daughter six weeks previously, and thought her "very smart and jolly;" and she noticed how fat she was getting, and how well she looked.

Her father, æt. 48, was said to be a strong, healthy man, who had had no serious illness but "a growth in the ear which got mixed up with the nerves and caused paralysis," and which was operated on four years ago.

Her mother, æt. 47, said she was always strong, and had had no serious illness. She had had nine children, all born alive and at full term; four or five very early miscarriages (six to eight weeks), but not in a continuous series. The children were all big and healthy except Charlotte, who was rather small at birth (but not shrunken and wizen-faced); the next child, two years older, was healthy, and there had been no intervening miscarriage. Of the nine children four had died in childhood, the others being healthy, except the eldest girl, aged 25, who is extremely anæmic.

Charlotte had appeared healthy and strong through childhood; she had scarlet fever at 8 years, but no other illness. Twelve months ago she had teeth extracted, and there was much bleeding, which the doctor said it was best not to stop. She menstruated at 14½, and at first this was "rather much," but her mother could not say whether this continued so.

Though she had been complaining as stated all the time she had been in the house, she did her work steadily and good-naturedly till at midday on Saturday, September 26th, she vomited, and said she had a "bilious attack."

She stayed in bed on the 27th and vomited once during the forenoon, but took her dinner. An hour or two after her manner became strange and apparently hysterical, she threw herself about, made grimaces at her mistress, and put out her tongue at her with her false teeth poised on the tip. Fearing for her sanity, the mistress sent hurriedly for advice; but the condition was so like hysteria that no serious view of the condition was formed at that time, and the friends were reassured concerning her.

On seeing her at about ten o'clock, however, I found her semi-comatose; the pupils contracted, and but slowly reacting to light; conjunctival reflex present, but much diminished; pulse 80, regular, but of a "slapping" quality, with a strong impulse quickly subsiding; respirations 23, and rather shallow.

She made no response on being spoken to, but turned slightly from a strong light; and on my attempting to remove her teeth there was too strong resistance by the muscles of the jaw for me to succeed in doing so; no effort at interference was made, however, by the hands.

There was possibly a little greater flaccidity of the muscles of the limbs of the right side than of the left, on both sides the knee-jerks were much exaggerated, and there was also very marked Babinski's sign in both feet. She had passed urine in the bed. There were no signs of middle-ear disease. Within half an hour the respirations became of an irregular Cheyne-Stokes character, and the pulse began to be slightly irregular. There was a slight blowing systolic murmur at the apex ; nothing abnormal detected in the abdomen.

At midnight Dr. Charles saw her with me, and by then the coma had considerably deepened, the pupils were dilated, conjunctival reflex was absent, slight divergent squint had appeared, and the pulse was more irregular and failing. The right optic disc was blurred and indistinct, knee-jerks both active, and Babinski's sign very marked.

There was no resistance offered to the removal of the teeth, the tongue and mouth appeared to be normal, and there was no abnormal odour to the breath.

Possibly the right side was a little more flaccid than the left, but no definite localising signs could be obtained, and though in the absence of an examination of the urine data were insufficient for a definite diagnosis, basal hemorrhage was considered as a probable cause of the condition.

Obviously no treatment would be of any avail, and none was attempted. The coma deepened ; convulsions, said by the nurse to have been frequent and general, supervened, and the girl died at 7.45 a.m. on the 28th.

REMARKS BY PROFESSOR I. WALKER HALL.

At the autopsy, made twenty-eight hours after death, the chief features were a diminished coagulability of the blood, a general hypoplasia of the circulatory organs, a large, round, gastric ulcer, and the basal hemorrhage. The subcutaneous tissues were very adipose. The liver, kidneys, intestines, lungs and sexual organs did not present any distinctive lesions.

The ulcer was situated on the anterior wall of the stomach, and had extended to the serous coat ; the edge of its left half was undergoing fibrosis, while that of its right half was clean cut.

The aorta was of small size both in its ascending and descending parts: the abdominal and cephalic arteries were thin walled, and contained fluid blood. The heart was small for the size of the body. The valves and orifices were quite normal, and there were no signs of recent or old peri-, endo-, or myo-carditis. No thrombi were present in any of the cavities.

On opening the skull, the basal hemorrhage was found. After removal of the brain, a large amount of fluid blood escaped from the severed arteries.

The hemorrhage appeared to originate from a small clot on the basal part of the right posterior cerebral vessels, and to extend via the pia-arachnoid and "iter" into the ventricles. The lateral ventricles both contained a small quantity of fluid blood, and the choroid plexus was very turgescent; from its surface fluid blood dripped continuously into the posterior horns of the lateral ventricles. No hemorrhages were found in the "pons" or "medulla." There were several cortical areas whose appearances were suggestive of "capillary hemorrhage."

Serial sections were made of the primary clot. A vessel with fibrous walls—apparently a venule or a fibrosed arteriole, it being impossible to determine which, although several differential stains were employed—opened into an aneurysmal dilatation. The opening was located definitely, and the continuity of the aneurysmal wall with the arterial coats was established. The contents of the saccular dilatation consisted of peripheral laminated clot, and a central area of red-blood cells. The part where rupture had occurred was quite evident.

The cerebral arteries were very thin, and the walls of some of the vessels varied in thickness and adventitial fibrosis. Neither atheroma nor endarteritis obliterans were present.

A careful search for syphilitic changes did not reveal any features of interest either in bones, lung, liver, spleen or eyes.

In the absence of other evidence, the lesions may be ascribed to some congenital defect in the blood apparatus. Cerebral hemorrhage may arise from arteries, capillaries, veins or aneurysm. In this case the absence of atheroma suggested that localised lesions, such as emboli, &c., might have led to the aneurysm.

None of these could be found. The condition of the blood, the appearance of the veins, the turgescence of the choroid plexus, all pointed to a venous, rather than to an arterial, cause. It seems probable, therefore, that the blood condition was the primary cause, and that the case should be classed under this head.

Had the case come under consideration twenty-four hours earlier, it would have been possible to carry out routine blood and other examinations. As it is, the record of the case is admittedly incomplete, and its mention only justified in view of some of its clinical features, and of the microscopical appearances demonstrated by the colour-photo-micrographs which were shown to the meeting.

The very fluid condition of the blood, the evidence of the length of time during which hemorrhage had been occurring, the extensive thinning and softening of the cerebral cortex, are all points of interest in the case, which make one regret that the patient had not come sooner under observation, and wish that more data had been obtainable for solution of some of the problems involved.

---

